# Early-onset drusen in Malattia Leventinese with *EFEMP1* mutation differ from drusen in age-related macular degeneration

**DOI:** 10.22336/rjo.2025.93

**Published:** 2025

**Authors:** Areeba Shakeel, Darshan Bhatt, Sarangapani Sripriya, Dhanashree Ratra

**Affiliations:** 1 Department of Vitreoretinal Diseases, Sankara Nethralaya, Chennai, India; 2 SNONGC Department of Genetics and Molecular Biology, Sankara Nethralaya, Chennai, India

**Keywords:** Early onset drusen, AMD, *EFEMP1* mutation, malattia leventinese, Doyne honeycomb dystrophy, ML = Malattia Leventinese, DHRD = Doyne honeycomb retinal dystrophy, AMD = Age-related macular degeneration, FAF = fundus autofluorescence, SSOCT = swept-source optical coherence tomography, MNV = macular neovascularization, *EFEMP1* = Epidermal growth factor-containing fibulin-like extracellular matrix protein 1, RPE = retinal pigment epithelium, BCVA = best corrected visual acuity, ORT = Outer retinal tubulations, VEGF = vascular endothelial growth factor, ONH = optic nerve head, ARG = Arginine, TRP = Tryptophan, EGF = Epidermal growth factor

## Abstract

**Purpose:**

To study the clinical, genetic, and phenotypic aspects of Malattia Leventinese (ML)/Doyne honeycomb retinal dystrophy (DHRD) and to differentiate it from age-related macular degeneration (AMD).

**Methods:**

Three cases of ML/DHRD from the Indian population were evaluated, including fundus examination, fundus autofluorescence (FAF), and swept-source optical coherence tomography (SSOCT). Genetic investigations involved screening for an inherited retinal gene panel using the Illumina MiSeq platform for one case. Pedigree charting, blood collection, DNA extraction, and variant annotation were performed, followed by pathogenicity assessment of the identified variants using multiple bioinformatics tools.

**Results:**

All cases exhibited early-onset central vision loss and small, radially distributed drusen, consistent with ML/DHRD. Genetic analysis done in patient one revealed a heterozygous, autosomal dominant, pathogenic mutation (c.1033C>T p.ARG345Trp) in the *EFEMP1* gene, confirming the ML diagnosis. Patient 1 had no late-stage complications, whereas patients 2 and 3 developed macular neovascularization (MNV). OCT showed gross thickening of the retinal pigment epithelium with hyperreflectivity, along with outer retinal tubulations (ORT) and interlaminar bridges, indicating outer retinal degeneration.

**Discussion:**

Malattia Leventinese is a rare autosomal dominant macular dystrophy caused by a single *EFEMP1* missense mutation (R345W), leading to early-onset radial or honeycomb drusen and central vision loss in the third decade. In this series, all patients showed typical radial drusen, with macular neovascularization in two cases, and demonstrated interlaminar bridges and ORTs on OCT. The mutant *EFEMP1* protein misfolds and accumulates abnormally between the RPE and Bruch’s membrane, accelerating drusen formation. Some phenotypic variability, including intrafamilial differences, likely reflects additional genetic or environmental modifiers. The presence of the R345W mutation, age at onset, and drusen distribution pattern are crucial for differentiating ML/DHRD from AMD.

**Conclusion:**

The identified pathogenic *EFEMP1* mutation (R345W) established a molecular link to ML/DHRD. Typical phenotypic patterns and drusen characteristics can differentiate ML/DHRD from AMD.

## Introduction

Malattia leventinese (ML)/Doyne honeycomb retinal dystrophy (DHRD) is a rare hereditary macular dystrophy characterized by a radially oriented drusen beneath the retinal pigment epithelium (RPE) [**1**]. ML has recently been classified as “Early Onset Drusen” (EOD), in which drusen-like deposits are observed at a younger age. The locus was mapped to chromosome 2p16-21, with a single missense mutation (R345W) (Arg to Trp) identified in the sixth epidermal growth factor domain of the fibulin-like extracellular matrix protein 1 (EFEMP1/S1-5, fibulin 3, FBNL3) as the leading cause [**2,3**].

*EFEMP1* was first isolated from fibroblasts from a patient with Werner syndrome, a premature-aging disease [**4**]. Unlike drusen in AMD, drusen in ML can often occur at the optic disc margin or nasal to it. Advanced stages result in geographic atrophy, pigmentary changes, and macular choroidal neovascularization (MNV) [**3**].

There are very few reports from Asia (**Table 1**). We present 3 cases from the Indian population, 1 with a confirmed mutation, and briefly review the literature, and discuss differentiation from the drusen observed in age-related macular degeneration (AMD).

**Table 1:** Review of literature on Malattia leventinese with genetic analysis

Previous study	Genetic mutation/Exon	Highlights
Heon et al. (1996) [**2**]	Short arm of chromosome 2	Chromosomal location of the gene involved in the pathogenesis
Stone et al. (1999) [**3**], Switzerland, the United States, and Australia	Heterozygous mutation Gene *EFEMP1*/10 c.1033C>T (Arg345Trp)	First report of a single non-conservative mutation found in both ML and DHRD
Matsumoto and Traboulsi (2001) [**5**], USA	Heterozygous mutation Gene *EFEMP1*/10 c.1033C>T (Arg345Trp)	This mutation remains the only cause of DHRD/ML/radial dominant drusen. Mutation absent in familial drusen
Narendran et al. (2005) [**6**], Australia	Found four previously described [287A>G, C>T, (ttg)9-12 repeat, Del T] and three novel sequence variations (29C>T, A>G, 112A>C)	The Arg345Trp mutation is the only mutation within either the coding or the adjacent intronic regions of the *EFEMP1* gene Other variations reported were unrelated to the disease
Takeuchi et al. (2010) [**7**], Japan	Heterozygous mutation Gene *EFEMP1*/10 c.1033C>T (Arg345Trp) found in all four patients	Novel haplotype found in one patient (8-T-G-C-M-T-G-A-T-G-3)
Zhang et al. (2014) [**8**] China	Heterozygous mutation Gene *EFEMP1*/10 c.1033C>T (Arg345Trp)	First report in a Chinese pedigree
Sheyanth et al. (2021) [**9**] Denmark	Heterozygous mutation Gene *EFEMP1*/10 c.1033C>T (Arg345Trp)	First Scandinavian case of molecular genetically verified DHRD/ML

## Methods and results

We report three patients with clinical features of malattia leventinese. Written informed consent was taken from each patient for examination, investigation, and use of material for research and publication.

### Case 1

**Fig. 1:**
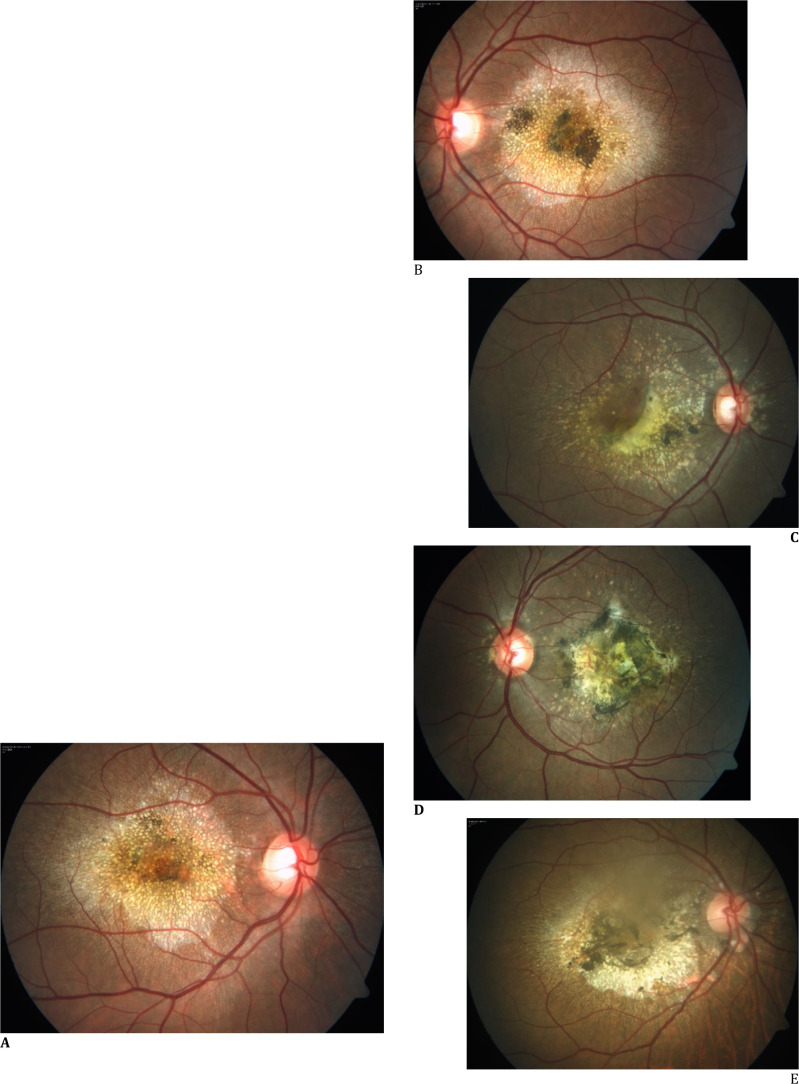

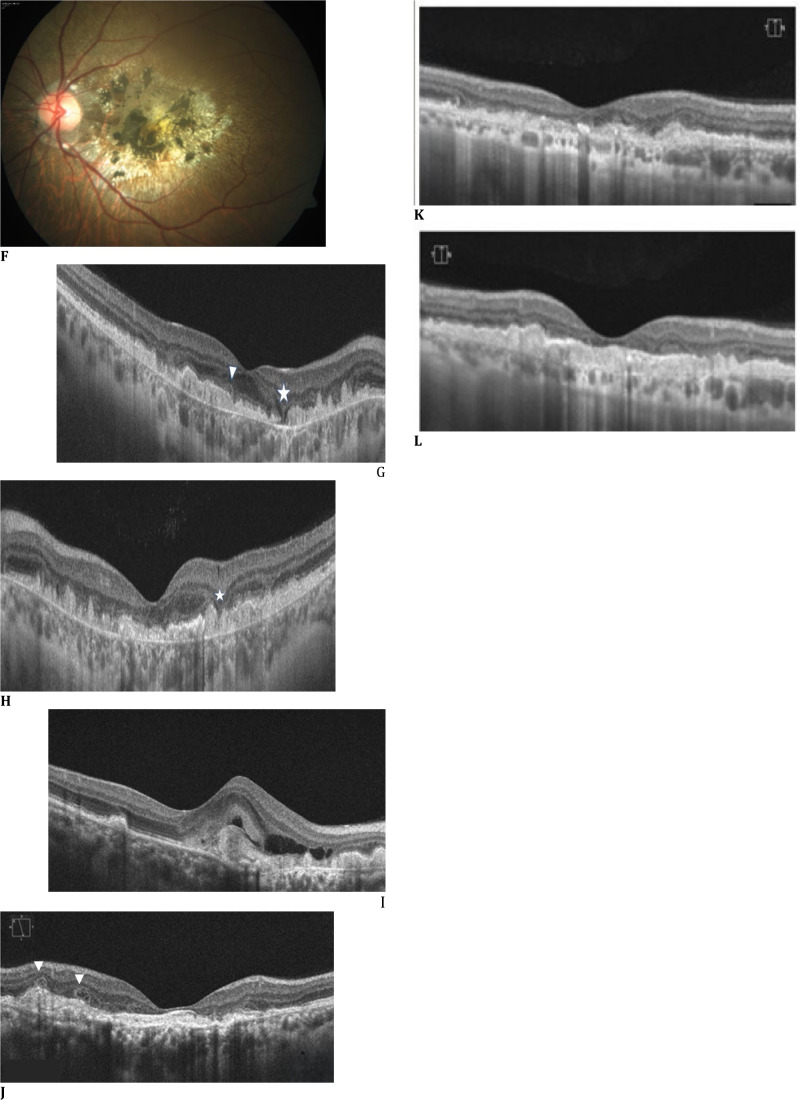
Color Fundus photo of patient 1(37/M) showing radially oriented drusen centered at macula with foveal sparing in right eye (BCVA 20/20) (**A**) and with pigmentary changes at fovea in left eye (BCVA 20/60) (**B**); Patient 2 (40/M) showing radial and peripapillary drusen in both eyes with active MNV in right eye (BCVA 20/60) (**C**) and scarred, pigmented neovascular membrane in left eye (BCVA 20/2000) (**D**); Patient 3 had radially arranged drusen with scarred MNV, pigmentary changes and peripapillary atrophy in both eyes (**E, F**); swept source optical coherence tomography of Patient 1 shows diffuse RPE atrophy causing hyper transmission, Sub RPE deposits causing saw tooth like elevation, interlaminar bridges (labelled with *) and outer retinal tubulations (labelled with arrowheads) in both eyes (**G, H**); patient 2 additionally had active type 1 MNV in juxtafoveal (inferonasal) area in the right eye (**I**) and scarring with foveal thinning in the left eye (**J**); patient 3 developed subfoveal scarring with retinochoroidal atrophy in both eyes (**K, L**)

**Fig. 2:**
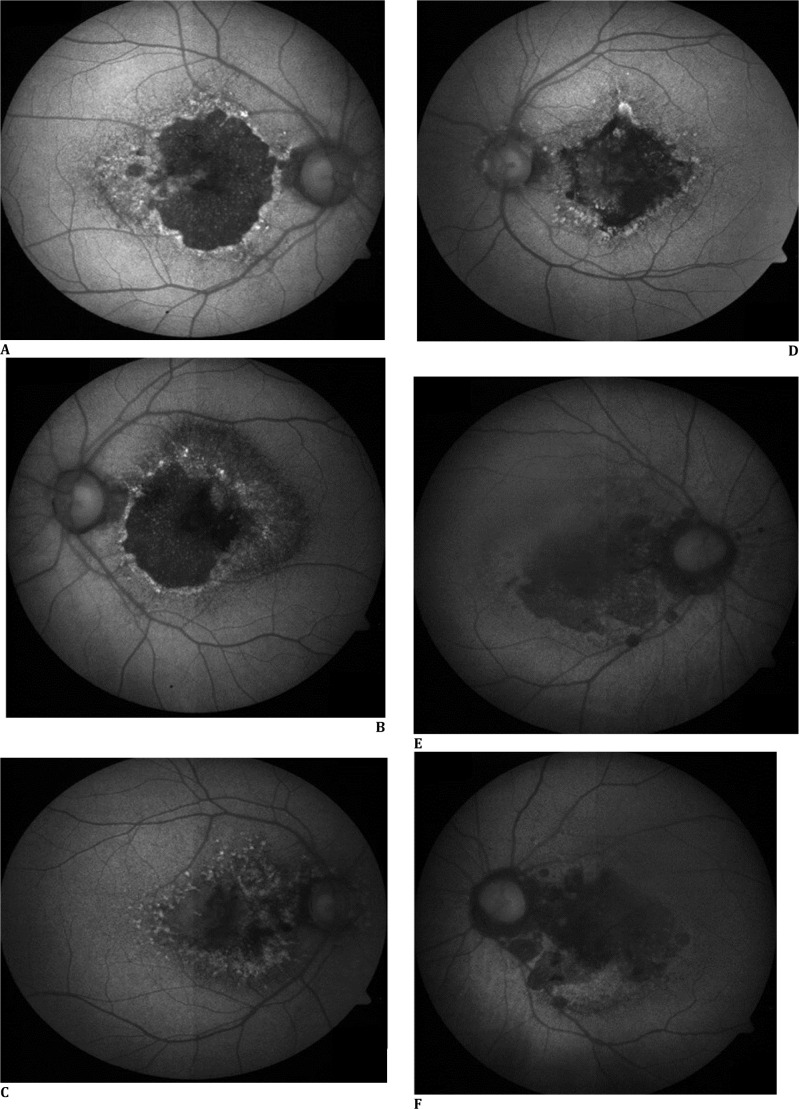
Fundus autofluorescence of patient 1 (**A, B**) shows hypoFAF at macula indicating RPE atrophy and rim of hyper FAF dots representing large drusen; in patient 2, hypoFAF at macula indicates scarring/RPE atrophy in left eye (**D**) and combination of active and scarred MNV in the right eye (**C**), surrounding rim of hyperFAF dots was also noted; patient three had hypoFAF in both eyes (**E, F**) which is in correlation with scarring and RPE atrophy at macula

A 37-year-old male with gradually progressive central vision loss over 6 years had best corrected visual acuity (BCVA) of 20/20 in the right eye and 20/60 in the left eye. Retinal examination showed numerous, small, radially oriented, yellowish-white, hard drusen radiating from the center. The left eye showed darkly pigmented patches in the center of the lesion. The foveal area in the right eye appeared to be spared (**Fig. 1A, B**). Fundus autofluorescence (FAF) showed a large confluent area of hypo FAF surrounded by a rim of tiny hyperFAF dots in both eyes, suggestive of RPE atrophy. The right eye showed a small strip of preserved RPE in the center (**Fig. 2A, B**). Swept-source optical coherence tomography (SSOCT) showed gross RPE atrophy with hypertransmission. A thick deposition of hyperreflective material was observed beneath the RPE layer, with multiple saw-tooth-like elevations. The photoreceptor layer was not visible, and the external limiting membrane (ELM) was disrupted in places. Outer retinal tubulations and interlaminar bridges were observed (**Fig. 1G, H**). The choroid was of normal thickness. Pedigree charting was performed, and 8 ml of blood was collected in an ACD Vacutainer, followed by DNA extraction using the NucleoSpin® Blood XL kit (Macherey-Nagel, GmbH, Germany). Screening for the inherited retinal gene panel was performed on the Illumina MiSeq platform by Medgenome Labs Ltd (Bangalore). Variations were annotated using the VEP tool at Medgenome by comparing with the 1000 Genome project [**10**]. The pathogenicity status of the variants was predicted online using bioinformatic tools (SIFT, PolyPhen, Mutation taster, Mutation assessor, and Variant Effect Predictor) and classified according to guidelines of the American College of Medical Genetics and Genomics (ACMG) [**11**].

A heterozygous, autosomal dominant, pathogenic mutation c.1033C>T p.ARG345Trp, comprising a C-to-T mutation resulting in an arginine-to-histidine change at codon 345 of *EFEMP1*, was identified (Clinvar ID RCV000726861). This variant has not been reported in the 1000 genomes and gnomAD Databases. The reference codon is conserved across species, and in silico predictions from SIFT, LRT, and MutationTaster indicate a damaging effect.

### Case 2

A 40-year-old man had poor vision, more in the left eye, for 4 years. The vision was 20/60 in the right eye and counting fingers at 1 m (20/2000) in the left eye. The retinal evaluation revealed typical small white radially oriented drusen in the macular area. Some drusen were also observed on the nasal side of the optic nerve head. An active MNV was observed in the right eye and a scarred MNV with hyperpigmentation in the left eye (**Fig. 1C, D**). SSOCT showed gross thickening of RPE and Bruch’s membrane, drusenoid pigment epithelial detachment (PED) in both eyes. Subretinal hemorrhage, subretinal fluid, intraretinal cystic spaces, and abnormal vasculature at the outer retina indicated active MNV in the RE, while old, mature, abnormal vasculature with foveal thinning and a hyperreflective subfoveal scar were observed in the LE (**Fig. 1I, J**). Malattia leventinese was diagnosed based on clinical examination and investigations. The patient received an anti-VEGF injection into the RE. The importance of genetic counseling and testing was explained, but the patient declined genetic testing.

### Case 3

A 49-year-old female had progressive loss of vision in both eyes for 7 years. Her visual acuity was 20/80 in the RE and 20/200 in the LE. Typical ML features of multiple small, discrete, radial subretinal drusen were observed around the macula, with hyperpigmentation and peripapillary atrophy, in both eyes (**Fig. 1E, F**). SSOCT showed retinochoroidal atrophy with subfoveal scarring (**Fig. 1K, L**). The importance of genetic counseling and testing was explained. She was advised to use low vision aids for visual rehabilitation.

## Discussion

DHRD or ML is a rare, hereditary, macular degenerative disease characterized by early-onset drusen with radial or honeycomb distribution, due to a single missense mutation in the *EFEMP1* gene [**3**]. In this series, all patients had a typical radial arrangement of drusen with early-onset central vision loss in the third decade, consistent with ML. Notably, case 1 exhibited no late-stage complications, while the other two cases developed MNV. Genetic analysis of case 1 revealed autosomal dominant inheritance and a pathogenic mutation in the *EFEMP1* gene, supporting the ML diagnosis.

Both small and large drusen are encountered with ML, along with pigmentary changes. Large drusen of ML are similar to drusen in AMD and show focal dome-shaped or diffuse deposition of hyperreflective material between the RPE and Bruch’s membrane. Conversely, small radial drusen are similar to early-onset cuticular drusen as irregular thickening of the RPE/Bruch’s membrane complex and sawtooth-like RPE elevations with preservation of neurosensory retina [**12**]. On OCT, interlaminar bridges appear as hyperreflective bands spanning from the outer to the inner retina, surrounded by hyporeflective bands **(Fig. 1G, H)**. These structures signify an early neuronal injury response, marked by hypertrophy and activation of Müller cells within the parafoveal region. They have been described in conditions like choroideremia and Bietti’s crystalline dystrophy, but not in ML/DHRD [**13**]. Outer retinal tubulations (ORT), observed as ovoid hyporeflective spaces with hyperreflective borders, are signs of photoreceptor loss and indicate poor visual prognosis. These have been reported in ARMD and other retinal disorders, but not in ML/DHRD. Case 1 in this series showed both interlaminar bridges and ORTs, indicating outer retinal degeneration, even though clinically the patient’s vision was unaffected. The large drusen were hyperautofluorescent, but small drusen and areas of RPE atrophy were hypoautofluorescent.

The observed variation lies in the calcium-binding EGF domain of the *EFEMP1* protein [PF00013], which contains 5-6 such domains. A single Arg-Trp (R345W) mutation alters the last domain of *EFEMP1* (similar to fibrillin mutations), leading to an aberrant accumulation of the protein between the RPE and Bruch’s membrane, creating a physical barrier and leading to drusen formation. The Arg345Trp mutation has been reported only in familial or sporadic early-onset drusen, not in age-related drusen such as AMD, or in familial drusen cases [**14**].

The wild-type *EFEMP1* is a secreted protein, whereas the mutant-type *EFEMP1* is a misfolded, inefficiently secreted protein that is retained within cells [**14**]. In normal eyes, *EFEMP1* is observed in the photoreceptor layer and inner retinal layers, but is conspicuously absent in the outer layers, namely, RPE-Bruch’s and choroid. However, in both ML and AMD eyes, mutant *EFEMP1* is found beneath the RPE overlaying sub-RPE deposits, but it does not appear to be a significant component of drusen [**14**]. The composition of drusen is almost similar in both diseases.

Variation in the phenotype of ML/DHRD can be attributed to other genetic factors or environmental factors. Phenotypic studies have demonstrated that this mutation also manifests marked intrafamilial phenotypic variability [**12,14**].

The R345W mutation may accelerate drusen formation in ML [**14**]. Hence, if this mutation is present, the pathogenic process would be faster and earlier, making ML diagnosis more appropriate than AMD. In the absence of a mutation, modifications induced by oxidative, thermal, or other stresses may cause denaturation of *EFEMP1*, leading to its accumulation in AMD eyes. The presence of the *EFEMP1* gene mutation, age of onset, pattern of drusen distribution, and precipitating factors can distinguish ML/DHRD from AMD. **Table 2** shows the differentiating features between ML and AMD. They have non-correlating genotype/phenotype relationships as observed between inherited and age-related conditions like Alzheimer’s disease and Parkinson’s disease.

**Table 2:** Features of drusen in age-related macular degeneration (AMD) and Malattia leventinese (ML)

Characteristics	Age-related macular degeneration	Malattia Leventinese
**Age of presentation**	5th decade or older	3rd/ 4th decade
**Occurrence**	One of the leading causes of central vision loss	Rare cause for central vision loss
**Laterality**	Bilateral	Bilateral
**Sex predilection**	M=F	M=F
**Inheritance**	Non-hereditary, age-related	Autosomal dominant
**Arg345Trp mutation in EFEMP1/Fibulin-3**	Not detected	Pathogenic
**Composition**	Amyloid P and Vitronectin	Amyloid P and Vitronectin
**Pattern of drusen distribution**	Mostly limited to the macula	Macula, peripapillary, and radially up to mid periphery
**Site of drusen accumulation**	Between RPE and Bruch’s membrane	Between RPE and Bruch’s membrane
**Histopathology**	No fixed pattern	Laminated appearance of deposits
**Immunoreactivity to anti-fibulin 3 and anti-collagen 4 antibodies**	Absent	Present
**Effect of risk allele (CFH/ ARMS2) on disease severity**	Yes	No

In advanced stages of ML/DHRD, treatments for neovascular AMD, such as photodynamic therapy or anti-VEGF antibodies, should be considered. Case reports have recently been published in Indian literature, but they lacked molecular diagnosis [**15**].

## Conclusion

In conclusion, typical clinical features of early-onset drusen, radially arranged around the fovea and ONH, are pathognomonic of ML. The *EFEMP1* mutation leads to early-onset drusen that differ distinctly from those in AMD. To our knowledge, this is the first report of this mutation from the Indian population.

## References

[1] Gelvez N, Hurtado-Villa P, Flórez S, Brieke AC, Rodríguez F, Bertolotto AM, Tamayo ML (2021). Diagnostic definition of malattia leventinese in a family from Colombia. Biomédica.

[2] Héon E, Piguet B, Munier F, Sneed SR, Morgan CM, Forni S, Pescia G, Schorderet D, Taylor CM, Streb LM, Wiles CD (1996). Linkage of autosomal dominant radial drusen (malattia leventinese) to chromosome 2p16-21. Archives of Ophthalmology.

[3] Stone EM, Lotery AJ, Munier FL, Héon E, Piguet B, Guymer RH, Vandenburgh K, Cousin P, Nishimura D, Swiderski RE, Silvestri G (1999). A single *EFEMP1* mutation associated with both Malattia Leventinese and Doyne honeycomb retinal dystrophy. Nature Genetics.

[4] Lecka-Czernik B, Lumpkin CK, Goldstein S (1995). An overexpressed gene transcript in senescent and quiescent human fibroblasts encoding a novel protein in the epidermal growth factor-like repeat family stimulates DNA synthesis. Molecular and cellular biology.

[5] Matsumoto M, Traboulsi EI (2001). Dominant radial drusen and Arg345Trp EFEMP1 mutation. American Journal of Ophthalmology.

[6] Narendran N, Guymer RH, Cain M, Baird PN (2005). Analysis of the EFEMP1 gene in individuals and families with early onset drusen. Eye.

[7] Takeuchi T, Hayashi T, Bedell M, Zhang K, Yamada H, Tsuneoka H (2010). A novel haplotype with the R345W mutation in the EFEMP1 gene associated with autosomal dominant drusen in a Japanese family. Investigative Ophthalmology & Visual Science.

[8] Zhang T, Xie X, Cao G, Jiang H, Wu S, Su Z, Zhang K, Lu F (2014). Malattia leventinese/Doyne honeycomb retinal dystrophy in a Chinese family with mutation of the EFEMP1 gene. Retina.

[9] Sheyanth IN, Lolas IB, Okkels H, Kiruparajan LP, Abildgaard SK, Petersen MB (2021). First reported case of Doyne honeycomb retinal dystrophy (Malattia Leventinese/autosomal dominant drusen) in Scandinavia. Molecular Genetics & Genomic Medicine.

[10] McLaren W, Pritchard B, Rios D, Chen Y, Flicek P, Cunningham F (2010). Deriving the consequences of genomic variants with the Ensembl API and SNP Effect Predictor. Bioinformatics.

[11] Watson M (2013). Incidental findings in clinical genomics: a clarification. Genetics in Medicine.

[12] Querques G, Guigui B, Leveziel N, Querques L, Bandello F, Souied EH (2013). Multimodal morphological and functional characterization of Malattia Leventinese. Graefe’s Archive for Clinical and Experimental Ophthalmology.

[13] Ratra D, Chattree S, Raviselvan M, Pradhan A, Giridhar S (2022). Structural and functional phenotypic features and molecular analysis of Indian patients with Bietti crystalline dystrophy. Indian Journal of Ophthalmology.

[14] Marmorstein L (2004). Association of EFEMP1 with malattia leventinese and age-related macular degeneration: a mini-review. Ophthalmic genetics.

[15] Thomas NR, Mohankumar A, Rajan M (2023). Malattia leventinese. Indian Journal of Ophthalmology-Case Reports.

